# Polymer induced liquid crystal phase behavior of cellulose nanocrystal dispersions†

**DOI:** 10.1039/d2na00303a

**Published:** 2022-10-06

**Authors:** Qiyao Sun, Viviane Lutz-Bueno, Jiangtao Zhou, Ye Yuan, Peter Fischer

**Affiliations:** Department of Health Science and Technology, ETH Zurich 8092 Zurich Switzerland qiyao.sun@hest.ethz.ch peter.fischer@hest.ethz.ch +41 44 632 9710; Paul Scherrer Institute 5232 Villigen PSI Switzerland

## Abstract

Cellulose nanocrystals (CNCs) are a promising bio-based material that has attracted significant attention in the fabrication of functional hybrid materials. The rod-like shape and negative surface charge of CNCs enable their rich colloidal behavior, such as a liquid crystalline phase and hydrogel formation that can be mediated by different additives. This study investigates the effect of depletion-induced attraction in the presence of non-absorbing polyethylene glycol (PEG) of different molecular weights in CNC aqueous dispersions, where the polymer molecules deplete the space around particles, apply osmotic pressure and drive the phase transition. Polarized light microscopy (PLM), rheology, small angle X-ray scattering (SAXS) and atomic force microscopy (AFM) are used to characterize the phase behavior over a time period of one month. In our results, pure CNC dispersion shows three typical liquid crystal shear rheology regimes and cholesteric self-assembly behavior. Tactoid nucleation, growth and coalescence are observed microscopically, and eventually the dispersion presents macroscopic phase separation. PEG with lower molecular weight induces weak attractive depletion forces. Tactoid growth is limited, and the whole system turns into a fully nematic phase macroscopically. With PEG of higher molecular weight, attractive depletion force becomes predominant, thus CNC self-assembly is inhibited and nematic hydrogel formation is triggered. Overall, we demonstrate that depletion induced attraction forces by the addition of PEG enable precise tuning of CNC self-assembly and phase behavior with controllable mechanical strength and optical activity. These findings deepen our fundamental understanding of cellulose nanocrystals and advance their application in colloidal systems and nanomaterials.

## Abstract 2

Depletion induced attraction forces by the addition of PEG has been demonstrated to enable precise tuning of CNC self-assembly and liquid crystal phase behavior with controllable mechanical strength and optical activity.

## Graphical abstract

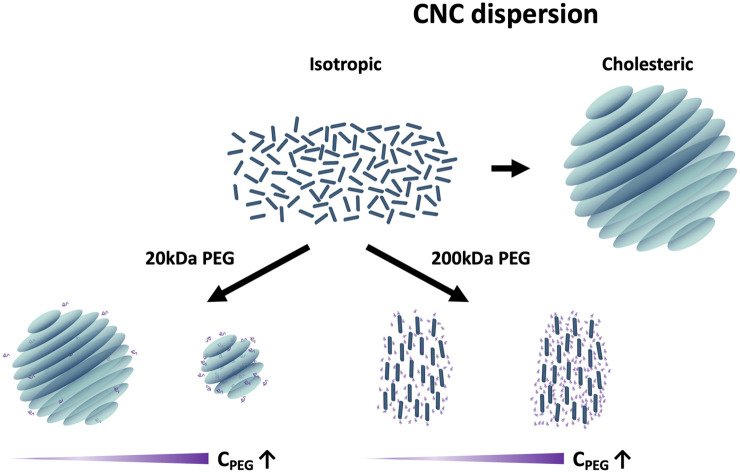

## 1 Introduction

**Fig. 1:**
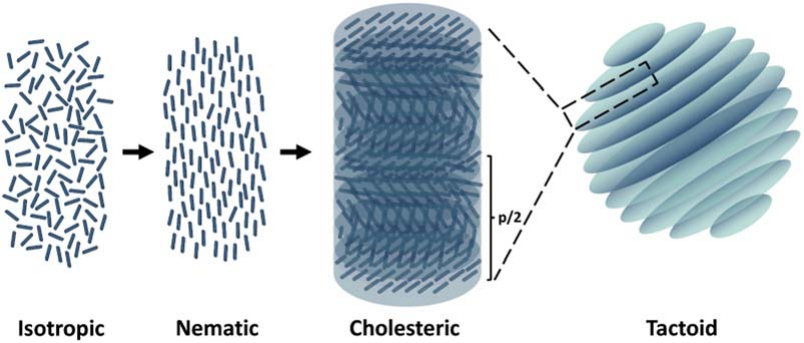
Schematic for nematic and cholesteric liquid crystal phase formation.

Nature is the most creative artist in designing complex hierarchical structures and serves as the best mentor in inspiring materials scientists. Cellulose, as the most abundant biopolymer, is regarded as a promising sustainable substitute for petroleum-based materials and has attracted attention because of diverse applications including biocompatible drug delivery systems,^1^ scaffolds for wound healing application,^2^ energy storage devices,^3^ or green stabilizers for Pickering emulsions.^4^ Cellulose nanocrystals (CNCs) are highly crystalline negatively charged rod-like particles obtained *via* sulfuric acid hydrolysis of wood pulp. Aqueous dispersions of CNCs show ability to form a cholesteric liquid crystalline phase, which is a type of liquid crystal with a helical structure and has chirality.^5,6^ Therefore, cholesteric liquid crystals are also called chiral nematic liquid crystals. As shown in Fig. 1, the CNC particles self-assemble into layers with orientational ordering, and the layer director axes vary between layers, which tends to be periodic. This periodic variation is characterized by pitch, *p*, which refers to the distance over which the liquid crystalline particles undergo a full 360° twist. Such a liquid crystal phase is formed due to energy competition according to the classic Onsager's theory. The orientational entropy that favors disorder competes with the excluded-volume entropy that favors order.^7–9^ The phase behaviors depend on the concentration,^10,11^ surface charge,^12^ and aspect ratio^13^ of the CNC particles and the ionic strength of the aqueous medium. While a low CNC concentration allows a random particle orientation and results in an isotropic phase, a high CNC concentration favors local orientational and positional ordering between single CNC particles. Experimentally, it is observed that the sample undergoes a phase transition from an isotropic to a cholesteric phase with increasing concentration. To be specific, the cholesteric phase nucleates from the isotropic phase in the shape of microdroplets (also named tactoids). First, dot-shaped small size tactoids without a finger print structure (homogeneous phase) are observed, and then homogeneous tactoids grow or merge into bigger ones, presenting the signature fingerprint structure (cholesteric phase).^11,14^

Polymers can modify the self-assembly and liquid–liquid phase behaviors of nanoparticle (NP) dispersions. Studies on polymer-induced liquid crystal phase behavior date back to the 1950s when Asakura and Oosawa first elucidated that the addition of a non-absorbing flexible polymer induces isotropic–nematic transition in dispersions of rod-like colloids.^15,16^ Depletion interaction was therefore defined, which depicts the attractive forces arising from the osmotic pressure difference in the regions depleted by polymers between colloidal particles and induces nematic phase formation of an original isotropic phase.^17,18^ A theoretical model of rod-like colloids with ideal polymers was established in the classic work by Lekkerkerker *et al.*^19^ Essentially, depletion interaction is positively correlated to both the depletant concentration and polymer chain length. Higher depletant concentration can result in change of osmotic pressure between colloidal particles, which hence drives the nematic phase transition observed experimentally. Experimental measurement of depletion forces using atomic force microscopy measurement was carried out by Biggs *et al.*, which confirmed that the attractive force increases with polymer molecular weight and concentration.^20^ On the other hand, polymer-induced gelation of CNCs is of interest in the application of injectable gels,^1^ raw materials for tissue engineering,^21^ and reinforce composites in 3D printing. Although polymer-induced CNC phase behaviors were predicted by numerous theoretical models and used in various applications, the bridge between them *i.e*. the microstructural evolution and the underlying mechanism of their phase transition are still unclear.

In this study, we demonstrate the depletion effect on CNC liquid crystal phase behavior induced by polymer addition. Non-absorbing neutral polyethylene glycol (PEG) of two different molecular weights (*M*_*w*_) was employed, and the induced phase transitions were observed over a time period of one month. Low-*M*_*w*_ (20 kDa) PEG induces changes of isotropic-nematic volume fractions with mediating tactoid size and growth speed at the microscale, while high-*M*_*w*_ (200 kDa PEG) induces gel formation with no tactoids but a bulk nematic domain. This enables the formation of adjustable viscoelastic, optically active hydrogels and provides possibility for comprehensive optical biomaterial fabrications. The transitions of optical properties and mechanical strength were characterized by polarized light photography/microscopy and rheological analysis, and the underlying structural changes were further determined by atomic force microscopy (AFM) and small-angle X-ray scattering (SAXS).

## 2 Results and discussion

### 2.1 Self-assembly and polymer-induced phase behaviors of CNC dispersions

**Fig. 2:**
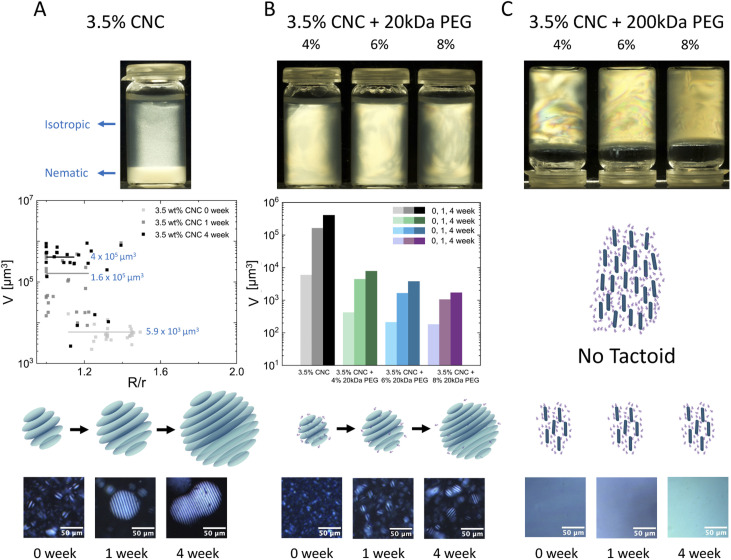
Macroscopic images, development of tactoid morphology (tactoid droplet volume *vs.* aspect ratio) over time, and microscopic observation of pure 3.5 wt% CNCs (A), 3.5 wt% CNCs with 4, 6 and 8 wt% 20 kDa PEG (B) and 3.5 wt% CNCs with 4, 6 and 8 wt% 200 kDa PEG (C).

Dispersions of CNCs in water with 20 kDa and 200 kDa PEG were prepared following the procedure described in the ESI.† Macroscopic visual inspection was conducted between cross-polarizers after 6 months of stabilization. As shown in the first row of Fig. 2, pure 3.5 wt% CNCs showed complete isotropic–nematic phase separation. No phase separation was observed in samples if polymers were added. 20 kDa PEG at different concentrations turned the mixtures into thorough nematic phases and all samples remained liquid-like. However 200 kDa PEG induced anisotropic hydrogel formation (the samples were turned upside down when photographed).

The development of tactoid (cholesteric liquid crystal droplets) morphology was monitored by PLM immediately after sealing sample dispersions, 1-week and 4-week stabilization, respectively. In the second row of Fig. 2, microscopic structural development of all sample combinations was tracked. The volume of tactoids against aspect ratio and time was traced by image analysis. Microscopic analysis allows measuring tactoid major (*R*) and minor (*r*) axes, and therefore their aspect ratio (*α* = *R*/*r*) and volume (approximated to the ellipsoid shape 
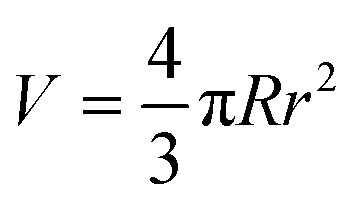
). Representative PLM images are shown in the third row. CNC colloidal stability and self-assembly behavior are in essence determined by the interplay between repulsive (electrostatic repulsion and steric hindrance) and attractive (van der Waals forces) interactions as described in DLVO theory.^22,23^ In this study, CNC nanorods self-assemble into a cholesteric liquid crystalline phase depending on the electric double layer and surrounding counterions in pure 3.5 wt% CNC water dispersion. Immediate cholesteric tactoid nucleation was observed, and then tactoids grew over time, while the aspect ratio decreased. These changes illustrate that the nematic phase transforms from homogeneous to cholesteric, which is in line with other liquid crystalline materials.^24,25^ Driven by gravity, the big tactoids collide, merge, sink and finally lead to macroscopic phase separation, as indicated in the first column of Fig. 2A.

The addition of 20 kDa PEG to the CNC dispersion delayed tactoid growth (Fig. 2B) and the effect was enhanced by increasing the PEG concentration. Similar to pure CNC dispersion, tactoid nucleation and growth was also observed in the mixtures with 20 kDa PEG, but the final size was significantly restrained. 20 kDa PEG addition resulted in lower final tactoid volume, which became more predominant at a higher PEG concentration. Different from the instant cholesteric tactoid formation of pure CNC dispersion, only minimum size homogeneous tactoids (dot-shaped, without a fingerprint structure, average volume *V̄* < 500 μm^3^) were observed in fresh samples with 20 kDa PEG. Small cholesteric tactoids were detected in 1-week samples of CNCs with 4 and 6 wt% 20 kDa PEG, while after 4 weeks in CNCs with 8 wt% PEG (precise size evolution shown in Fig. S2†). In addition, the cholesteric pitch seems to be independent of the 20 kDa PEG addition and its concentration (Fig. S3†). The same *p*/2 value as pure CNC dispersion at the corresponding time point was observed for all PEG concentrations. This could be attributed to the weak depletion effect imposed by 20 kDa PEG. When the space around CNC nanorods is depleted by 20 kDa PEG polymers, attractive force is induced, but electrostatic repulsion between CNCs persists and dominates. As shown in Fig. 2B, small size tactoids (homogeneous or 2–3 stripe cholesteric tactoids) are formed but “frozen” in the surrounding isotropic phase, therefore a continuum liquid crystalline phase is obtained. A slight decrease of *p*/2 was also observed over time, and this may be due to CNC surface charge loss as time passes.

However, in samples with 200 kDa PEG, no tactoid but only a nematic phase was observed. No obvious change was detected over time, *i.e*. the samples have shown transient stability (Fig. 2C). Samples with 6 and 8 wt% 200 kDa PEG addition are shown in Fig. S4.† In this case, depletion induced attractive force overcomes electrostatic repulsive force. Consequently, CNC particle mobility is prohibited and tactoid formation is restrained.

As demonstrated above, the weak depletion force deployed by non-absorbing 20 kDa PEG avoids phase separation and leads to a bulk nematic phase, in which CNC nanorods are depleted and assemble into smaller but numerous tactoids compared to pure CNC suspensions. With increasing PEG concentration, the depletion effect becomes more predominant, and smaller size tactoids are formed. However, the overwhelming depletion attractive forces applied by 200 kDa PEG cause tactoid disruption and induce hydrogel formation. The higher molecular weight of PEG has higher radius of gyration (*R*_g_) hence higher attractive force, which leads to stronger depletion.^19^ The resulting attractive interactions affect the spatial distribution and cage CNC nanorods in nematic ordering.^26^

Polymer induced depletion has been well established in the literature with different theoretical models,^27–29^ and composite photonic thin films have also attracted extensive attention.^30–32^ On the contrary, the microstructural evolution of such colloidal systems has been poorly understood. Herein, we use CNCs and non-charged polymer PEG as a model system, and reveal the structural evolution mediated by the molecular weight and concentration dependent depletion effect. Such systematic characterization provides more possibility and flexibility in manipulating microstructures for designing desired optical properties of CNC polymer hybrid systems.

### 2.2 Rheological characterization of polymer-induced phase behavior

**Fig. 3:**
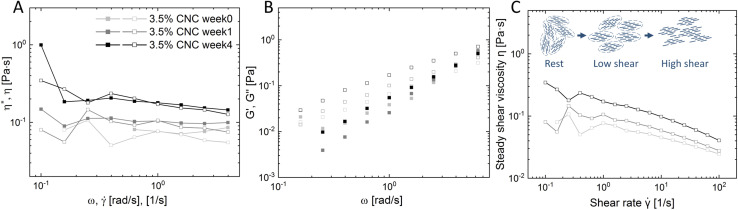
Time-series Cox–Merz rule rheological analysis: (A) valid regime of complex viscosity (η*, solid) and steady shear viscosity (*η*, empty) *versus* angular frequency (*ω*) and shear rate (*γ̇*) (detail inertia and minimum torque boundary calculation in the ESI†); (B) dynamic storage (*G*′, full) and loss modulus (*G*′′, empty) *versus* angular frequency (*ω*); (C) steady shear viscosity (*η*) *versus* shear rate (*γ̇*) of pure 3.5% CNCs at 0 week, 1 week and 4 weeks.

Rheology is applied to characterize CNC nanorods in the liquid crystalline state.^33^ The Cox–Merz rule has been applied here, which states that the complex viscosity (η*, Pa s) and the steady-shear viscosity (*η*, Pa s) will coincide when plotted against angular frequency (*ω*, rad s^−1^) and shear rate (*γ̇*, s^−1^), respectively. However, the presence of a liquid crystal or hydrogel structure will cause deviation from the Cox–Merz rule. Fig. 3 depicts the Cox–Merz rule comparison of pure 3.5 wt% CNCs. The accessible range of Cox–Merz comparison of η* *vs. η*, and *G*′ *vs. G*′′, and evolution of steady shear viscosity over time are illustrated in Fig. 3A–C, respectively.

Fig. 3A presents η* and *η* as a function of *ω* and *γ̇*. Both complex viscosity and steady shear viscosity increased over time, which indicates that more structures are formed, in line with the microscopic observation. The tactoid size increased over time, *i.e.* more localized structures formed, which enhances the resistance to flow, and therefore an increase of viscosity was detected. In addition, it was observed that at high shear rates and in the high angular frequency regime, η* and *η* were slightly diverging. The reason for this divergence is that in steady shear the self-assembled structure gradually destructed with increasing shear rate, while the small amplitude oscillation test does not break the structure.^34^ On decoupling η* into *G*′ and *G*′′ (Fig. 3B), the frequency sweep tests showed that viscoelasticity arose from the CNC ordering and developed over time. A viscoelastic liquid (*G*′ < *G*′′) behavior was detected, which is in good agreement with observations by Bertsch *et al.*^35^ and Xu *et al.*^36^ in pure CNC dispersions at intermediate concentrations.

Furthermore, a trend of steady shear viscosity was observed as shown in Fig. 3C, confirming the existence of liquid crystalline domains alone by steady shear viscosity. The steady shear viscosity curve obtained from isotropic dispersions usually only contains two regions: a constant viscosity plateau at low shear rates and a following rapid shear thinning region at high shear rates. However, liquid crystal systems may show an additional shear thinning region at low shear rates before reaching the plateau. This complex flow behavior is essentially due to the polydomain structure in LC systems. CNC nanorods in single domains have a local liquid crystalline ordering (tactoids), and these domains are surrounded by an isotropic background, where particles are randomly aligned. However, the direction of each domain is random without an alignment. In the low shear rate regime, the adjacent isotropic bulk starts to flow. In the meantime, the tactoids containing internal alignments only collide, deform or expand. These re-orientations and deformations oppose the shear flow and show an apparent rheological shear-thinning behavior.^37,38^ With increasing shear these domains (tactoids) orient along the shear flow, entering the relative plateau (Regime II). At high shear rates, LC alignments are finally destroyed and all particles are forced into the flow direction (Regime III). This three-regime behavior may differ from one LC system to another, but relatively commonly observed in CNC intermediate concentration dispersions.^34,39–41^ In our study, a plateau with fluctuations at the beginning of the low shear regime and clear shear-thinning in the high shear regime was obtained instead of three distinct regimes. This can be attributed to the concentration of the co-existing regime used in this study, therefore the 3 regimes are not as distinct as pure nematic phase dispersion. The polydisperse nature of CNCs may also play a role, since the resulting broader biphasic region than theoretical prediction could disseminate the initial viscosity drop across the whole biphasic region.^40^

**Fig. 4:**
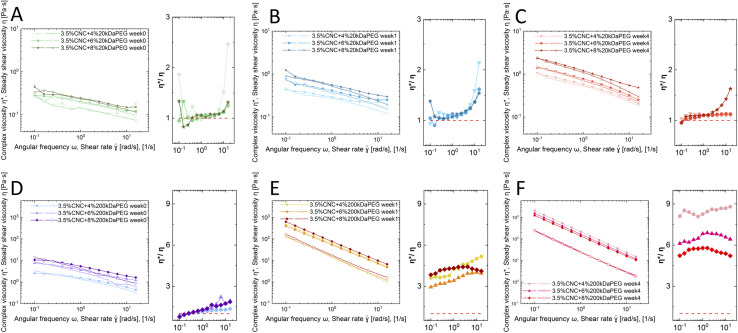
Time-series Cox–Merz rule rheological analysis: 3.5 wt% CNCs with 4% 20 kDa PEG, 6 wt% 20 kDa PEG and 8% 20 kDa PEG at 0 week (A), 1 week (B) and 4 weeks (C); 4% 200 kDa PEG, 6 wt% 200 kDa PEG and 8 wt% 200 kDa PEG at 0 week (D), 1 week (E) and 4 weeks (F).

Cox–Merz rule validation was conducted as well for the dispersions with polymer addition (4, 6, 8 wt% 20 kDa PEG and 200 kDa PEG). A subplot of ratio between η* and *η* was added besides the main Cox–Merz plot for better visualization of the deviation. The 20 kDa PEG samples slightly showed a Cox–Merz rule deviation (Fig. 4A–C), but the overall rheological behavior remained intact. At 0 and 1 weeks, the ratio of η* and *η* fluctuated around 1, whereas a clear deviation was detected at week 4. Both complex viscosity η* and steady shear viscosity *η* increased over time, and were slightly higher compared to pure CNC dispersion. The system remained as a viscoelastic liquid (*G*′′ > *G*′, Fig. S5A–C†). Similar to pure CNC dispersion, the liquid crystalline phase is confirmed by the three-regime behavior under steady shear flow (empty symbol in Fig. 4A–C). This is in line with the visual observation, that 20 kDa PEG converts biphasic dispersion (isotropic + nematic) into monophasic (only nematic, where tactoids are surrounded by isotropic background). Tactoid formation was observed, but the size is restrained by the polymer addition. Meanwhile the network is strengthened by the induced depletion attraction force, with the electrostatic repulsion playing the dominant role.

On the other hand, the 200 kDa PEG converted the system into a hydrogel. This is confirmed by clear Cox–Merz rule failure (Fig. 4D–F), where the ratio between η* and *η* showed significant deviation from 1 and this effect became more pronounced over time. The hydrogel showed a distinct increase in η* over a week and a slight further increase after 4 weeks, showing that a more dense structure is formed during aging. This is further confirmed by the viscoelastic solid behavior (*G*′ > *G*′′ shown in Fig. S5D–F†). While all fresh samples with 200 kDa addition stayed liquid-like, *i.e. G*′′ > *G*′ (Fig. S5D†), a gel behavior is observed at 1 week and 4 weeks inspection, *i.e. G*′ > *G*′′ (Fig. S5E and F†). Further, both *G*′ and *G*′′ showed a slight increase from 1 week to 4 weeks. The attraction gradually turns so strong that CNC nanorods are tightly arrested. Yet, this effect is independent of concentration. The depletion attraction generated by the applied polymer concentration lower limit is already beyond the maximum attraction interaction that can be induced, therefore no further network strength enhancement is observed.

### 2.3 Probing polymer-induced CNC structural evolution using SAXS and AFM

**Fig. 5:**
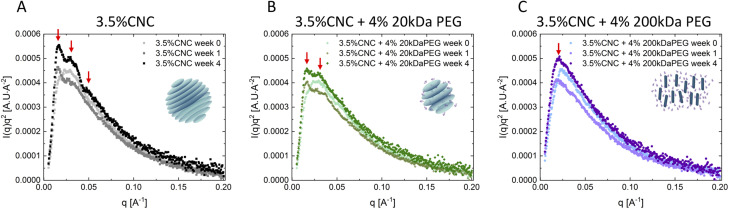
Time-series Lorentz-corrected scattering intensities as a function of scattering vector *q* of pure 3.5 wt% CNCs (A), 3.5 wt% CNCs with 4 wt% 20 kDa PEG (B), 3.5 wt% CNCs with 4 wt% 200 kDa PEG (C) at 0 week, 1 week and 4 weeks.

The nanostructural evolution of the CNC dispersions and mixtures was investigated using SAXS and AFM. Fig. 5A–C show the Lorentz-corrected SAXS curves of CNC dispersions without and with 4 wt% 20 kDa and 200 kDa PEG addition at 0, 1 and 4 weeks. A structural ordering arising from CNC self-assembly was detected in pure CNC dispersion (Fig. 5A): only one broad peak in week 0 fresh samples (*q* ∼ 0.023 Å^−1^), followed by secondary (*q*_1_ ∼ 0.016 Å^−1^, *q*_2_ ∼ 0.028 Å^−1^) and tertiary (*q*_3_ ∼ 0.047 Å^−1^) peaks appearing at 1 and 4 week samples respectively, indicating more ordered structures are formed over time. A fixed ratio (1 : 1.75) of the secondary order peaks was observed, which is regarded as a signature of tactoids in scattering curves.^42,43^ The two characteristic peaks correspond to the two characteristic distances in the cholesteric phase, namely the interparticle distance within a single cholesteric plane and the interplane distance between cholesteric planes. Homogeneous nematic tactoids with only orientational order are first nucleated from the isotropic phase, designated by a single characteristic distance. CNC particles within one plane orientate in the same direction with a certain interparticle distance. During growth of tactoids, the cholesteric planes start to pile up and twist at a certain angle meantime to minimize the electrostatic repulsion force. This induces complex positional order, and also leads to the signature stripe texture of the cholesteric tactoids, corresponding to the secondary peaks of the scattering curve.^24,44^ 20 kDa PEG addition only modified SAXS curves slightly. Only one broad peak (*q* ∼ 0.027 Å^−1^) was observed in week 0, followed by secondary peaks (*q*_1_ ∼ 0.016 Å^−1^, *q*_2_ ∼ 0.028 Å^−1^) in 1-week samples. It is noted that, although the correlation peaks became broader with polymer addition, the position of the two secondary order peaks remained unchanged, proving that the two characteristic distances, thus the cholesteric structure stayed intact. A third peak was only detected in pure CNC dispersion after 4 weeks. We argue that this might arise from exotic structures formed (*e.g.* cholesteric bulk) in pure CNC dispersion, but this is inhibited by attractive force in polymer added samples. With 200 kDa PEG addition, SAXS curves changed significantly and only exhibited one peak (*q* ∼ 0.027 Å^−1^) in the low *q* regime without structural development over time. This is indicative of a gel structure.^45^ The mobility of CNC particles is significantly reduced by depletion attraction, whereas the electrostatic repulsion between CNCs resists aggregation. Cholesteric phase formation is inhibited but the nematic ordering is maintained. The SAXS results are in agreement with our microscopic observation. The characteristic ratio of secondary order peaks was only observed in pure CNCs and with low-*M*_*w*_ PEG dispersions, which are exactly the case where tactoid formation was observed microscopically. Meanwhile, the formation of a nematic hydrogel under PLM is also consistent with the indication of a single SAXS peak in CNC dispersions with high-*M*_*w*_ PEG. The pure polymer solutions were also analyzed by SAXS and the results confirmed that PEG has no contribution to the ordering (Fig. S6†).

**Fig. 6:**
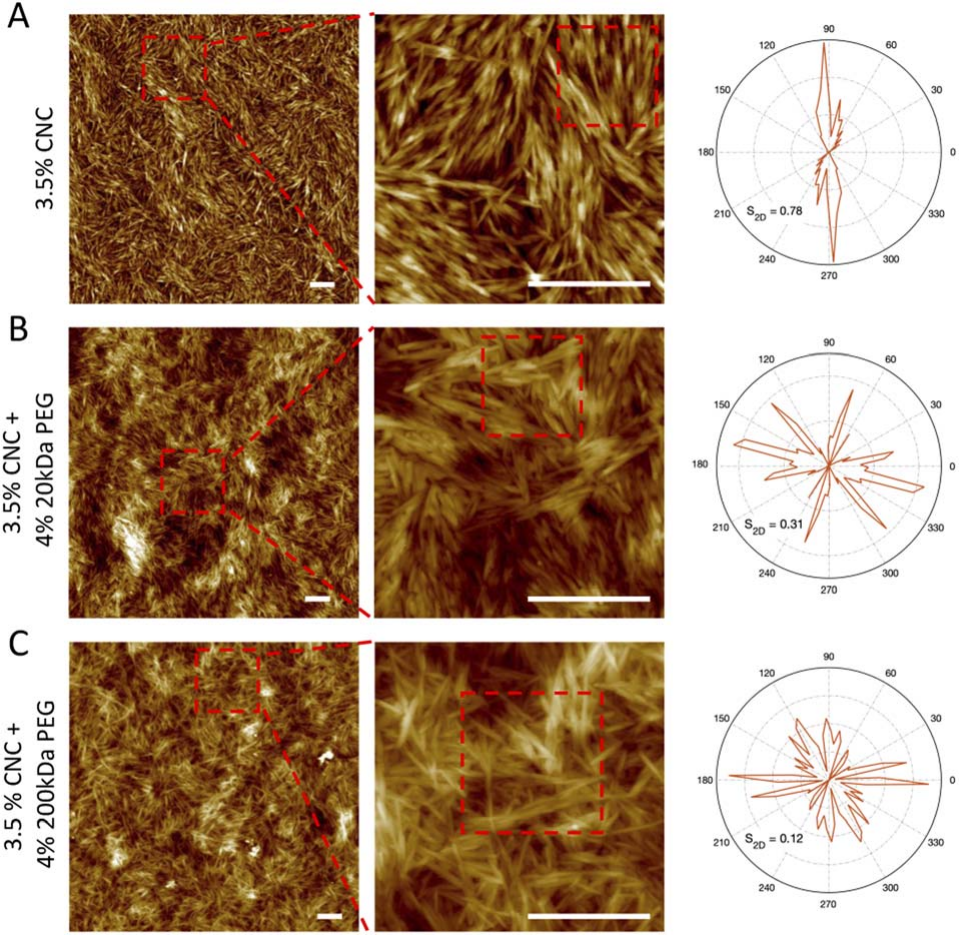
AFM images and orientation distribution of contour segments within selected areas of pure 3.5 wt% CNCs (A), 3.5 wt% CNCs with 4 wt% 20 kDa PEG (B), 3.5 wt% CNCs with 4 wt% 200 kDa PEG (C) at 1 week. Scale bar: 500 nm.

The structural difference and orientation distribution at the single particle level were visualized by AFM imaging. 1-week pure CNC dispersion with 4 wt% 20 kDa and 200 kDa PEG samples were selected as representatives. In the case of pure CNC dispersion (Fig. 6A), the CNC nanorods showed a clear localized orientation (selected area in the middle column), where they represented overall one orientation, which indicates a strongly ordered nematic domain. This is possibly associated with the cholesteric planes within tactoids, where CNC nanorods have the same orientational ordering in one plane. Therefore, after deposition onto mica, “clusters” of particles are formed by the cholesteric planes. The presence of 20 kDa PEG slightly disturbed the alignment with showing mainly triple orientation. This is in line with our previous observation of smaller size tactoids, where weak attractive interactions inhibit longer range alignments (*i.e.* big size tactoids), but the CNC nanorod cholesteric ordering within tactoids is maintained since repulsive electrostatic interaction still plays the predominant role (Fig. 6B). On the other hand, with 200 kDa PEG addition, CNC particles showed random alignment, possessing multiple directions in the orientational distribution map (Fig. 6C). This confirms that the depletion induced attractive force overcomes the electrostatic repulsion. CNC particles do not have the propensity to assemble into cholesteric ordering but are arrested in a gel form. The nematic ordering was not captured by AFM images, possibly due to the sample preparation, especially transferring sample dispersion onto the mica substrate. The homogeneous nematic ordering might easily rearrange and randomly deposit on the substrate, whereas the former cholesteric phase would be preserved in the tactoid and then vividly shown in AFM images after deposition. The order of the assembly is also quantified by the 2D order parameter *S*_2*D*_, which is defined as *S*_2*D*_ = 2〈 cos^2^*θ*_*n*_〉 − 1, where *θ* is the angle between the segments and the local director in the chosen area. The lower *S*_2*D*_ is associated with higher ordered alignment. Therefore, a distinct trend was detected: 0.78, 0.31 and 0.12 for pure, with 20 kDa PEG, and with 200 kDa PEG CNC dispersions respectively. This reassures that CNC self-assemblies are less aligned due to stronger attractive depletion forces.

## 3 Conclusion

As anisotropic charged rod-like nanoparticles, CNCs have received broad interest in designing complex functional materials because of the rich phase phenomenon induced by different additives and environmental stimuli. In this study, we have comprehensively investigated the optical and mechanical properties and nanostructural evolution upon polymer induced depletion attraction. CNC self-assembly has been tracked *via* SAXS in pure water dispersion at biphasic concentrations over one month. A fixed correlation peak ratio between secondary peaks was detected, indicating the formation of a cholesteric phase. Repulsive electrostatic interactions dominate CNC particles' assembly behavior, namely the isotropic – homogeneous – cholesteric transition. The structure evolved from random orientations to complex structures with orientational and positional orders. The whole dispersion exhibited a typical 3-regime shear rheology behavior. When slight depletion attraction has been applied by 20 kDa PEG addition, electrostatic repulsion stays predominant with the 2 signature SAXS correlation peaks. Tactoid size and more complex ordering are limited, with a fully cholesteric phase being observed. The system remains a viscoelastic liquid with slightly enhanced mechanical strength, which is enhanced by a higher concentration. This enables the possibility to fine-tune the liquid crystal structure at the micro-scale. While depletion induced gel presents only a nematic phase under polarized light upon addition of 200 kDa PEG concentration. In this case, overwhelmed depletion attractive force overcomes electrostatic repulsion, and the mobility of CNC particles has been significantly reduced and arrested in the nematic ordering state. The whole system appears as a nematic hydrogel with elastic solid behavior and Cox–Merz rule deviation. Our study demonstrates a simple approach to induce rich CNC dispersion phase behaviors by adding polymers, and possibly expands the design of complex biomaterials with adjustable optical activity and mechanical properties and self-assembled materials for bionanotechnology.

## Conflicts of interest

The authors declare no conflict of interest.

## Supplementary Material

NA-004-D2NA00303A-s001
