# Supplementary material for: Polymer induced liquid crystal phase behavior of cellulose nanocrystal dispersions
Source: Nanoscale Adv. 2022 Oct 6;4(22):4863–70. doi: 10.1039/d2na00303a (PMC9642361; doi:10.1039/d2na00303a)
Supplement: NA-004-D2NA00303A-s001 [file NA-004-D2NA00303A-s001.pdf]

Electronic Supplementary Material (ESI) for Nanoscale Advances.
This journal is © The Royal Society of Chemistry 2022

Support Information

Polymer Induced Liquid Crystal Phase Behavior of Cellulose Nanocrystal Dispersions

Qiyao Sun,[*,†] Viviane Lutz-Bueno,[‡] Jiangtao Zhou,[†] Ye Yuan,[†] and Peter Fischer[*,†]

†*Department of Health Science and Technology, ETH Zurich, 8092 Zurich, Switzerland*

‡*Paul Scherrer Institute, 5232 Villigen PSI, Switzerland*

E-mail: qiyao.sun@hest.ethz.ch (Q.S); peter.fischer@hest.ethz.ch (P.F)

Table of contents

- Experimental methods 1-2
- Supplementary Text 2-4
- Supplementary Figure S1-S6 5-7

Experimental methods

Materials. Polyethylene glycol (PEG, Mw = 20 and 200 kDa) was purchased from SigmaAldrich (Buchs, Switzerland). All chemicals were used as received without further purification. Milli-Q water was obtained from a Merck Millipore System (Darmstadt, Germany).

Characterization of CNC. CNC was kindly provided by CelluForce (Montreal, Canada).

The CNC particles are 78.59 ± 6.12 nm in length and 4.75 ± 0.05 nm in height, as determined by atomic force microscopy.[1] The critical volume fraction ϕ_c was approximated from an adaption of Onsager's approach[2] accounting for the charged double layer of CNC. The detailed calculations were done by Bertsch et al.[3]

Preparation of Pure CNC Dispersion and CNC Dispersions with Polymers. CNC crystallites were dispersed in Milli-Q water under magnetic stirring overnight, and the dispersions were further sonicated by a Hielscher UP200S (Teltow, Germany) at 160 W to a total energy input of 5000 J/g CNC to provide full dispersion. 3.5 wt% was chosen within co-existence for better microscopic phase transition observation. Polymers were added as powder, magnetic stirred overnight, followed by shortly sonication to prevent CNC aggregation. All followed characterization (except for AFM) was conducted immediately (also denoted as week 0), after 1 week and 4 weeks equilibrium.

Polarized Light Photography/Microscopy. The macroscopic features of liquid crystalline ordering in the samples were first checked between cross-polarizers to determine the presence of birefringence. Samples were loaded in 10 mL flasks and photographed with a Canon EOS 550D equipped with a 100 mm macro lens (Tokyo, Japan) after 6 month equilibrium. To confirm the macroscopic observation and analyze the development of the liquid crystal structures, samples were filled in glass cuvettes (0.2 × 4 × 40 mm^3, VitroTubes, Vitrocom) sealed with UV glue (Bondic, US) and imaged with polarized light microscope (PLM).

Rheology. Rheological measurements were performed on a Physica MCR 501 rheometer (Anton Paar, Graz, Austria) equipped with a Couette CC17 geometry. Frequency sweeps were performed from 0.1 - 100 rad/s in the linear viscoelastic regime, followed by steady shear experiments

performed from 0.1 - 100 1/s to evaluate the built-up structure by applying Cox-Merz rule. Instrument intertia effect and minimum torque boundary were added to exclude invalid data (Calculation details in Supplementary Information, see Fig. S1). For 1 week and 4 weeks samples, 6-hour was given for re-equilibrium after filling into the Couette cup before measurements. Experiments were performed at room temperature and samples covered by a tetradecane oil film to prevent water evaporation.

Small Angle X-Ray Scattering. Small angle X-ray scattering (SAXS) experiments were conducted using a Brucker AXS Micro XRF. The microfocused X-ray source was operated at a voltage and filament current of 50 kV and 1,000 μA, respectively. The measurements were performed with Cu Kα radiation (λ = 1.5418 Å) collimated by a 2D Kratky collimator and the spectra were collected by a Pilatus 100K detector. The scattering intensity *I(q)* was recorded in the interval of 0.005 < q <0.20 Å^{-1} where the scattering vector is defined as $q = \frac{4\pi}{\lambda} sin\theta$, with 2θ and λ being the scattering angle and wavelength. The experiments were conducted under vacuum at ambient temperature. Samples were sealed in 2 mm quartz capillaries and aligned in the X-ray beam.

Atomic Force Microscopy. An aliquot of CNC dispersions (3.5 wt% CNC, 3.5 wt% CNC with 4 wt% 20 kDa PEG, and 3.5 wt% CNC with 4 wt% 200 kDa PEG dispersions), prepared one week before imaging, were deposited without dilution on freshly cleaved mica for 1 min, followed by a smooth rinsing of Milli-Q water and a very gentle flow of compressed air to minimize artifacts. AFM measurements were performed by a Bruker multimode 8 scanning probe microscope (Bruker, USA) with an acoustic hood to minimize vibrational noise. AFM imaging was operated in soft tapping mode under ambient condition, using a commercial silicon nitride cantilever (Bruker, USA) with a vibration frequency of 70 kHz and a spring constant of 0.4 N/m. A relatively soft tip-sample interaction was applied during the AFM tip scanning.[4] AFM images were flattened using Nanoscope 8.1 software (Bruker, USA) and no further image processing was applied. The statistical analysis of orientation distribution of CNC segments was carried out using the FiberApp software.[5] Briefly, each CNC nanorod was traced along the contour and quantified the 2D origination by $S_{2D} = 2 < cos^2\theta_n > -1$, which is the angle between the segments and the local director in the chosen area.

Minimum torque and instrument inertia[6,7]

In shear rheology, material properties are obtained by applying force (torque) and measuring the deformation or deformation rate (or vice versa). In practice, the calculation of true stress and strain may be hampered by many factors such as instrument resolution and instrument inertia. These artifacts can result in apparent shear-thinning, shear-thickening, and viscoelastic responses while the material does not actually have these properties, especially in the context of biological material due to sample softness.

The minimum torque is typically the most important limitation for soft biological materials, which is normally specified by instrument manufacturers. The condition for acceptable data that measured torque is above some minimum limit, $T > T_{min}$. Experimental artifacts can also be caused by instrument inertia under transient conditions, where some torque is also associated with acceleration and deceleration of the measuring geometry, especially oscillatory tests at high frequency. The "Material torque" must exceed the "instrument inertia torque" in order to obtain reliable data. Herein, minimum torque and instrument inertia effects are controlled in

measurement of 3.5 wt% CNC liquid-crystal dispersion, experimental limitations are drawn in Figure S1. The calculation details are as follows:

- Minimum shear viscosity

Acceptable data is under the condition:

$$\eta > \frac{F_\tau T_{min}}{\dot{\gamma}} \quad (1)$$

where η is the viscosity, $\dot{\gamma}$ is the shear rate, T_{min} = $1 nN{\cdot}m$ is the minimum torque (instrument specification). The geometry factor F_τ for Couette is

$$F_\tau = \frac{\pi}{2R^2L} \quad (2)$$

where R and L are radius and height of the measuring cell.

- Minimum G and minimum η^*

$$G > \frac{F_\tau T_{min}}{\gamma_0} \quad (3)$$

where G is shear moduli (can be G' or G''), and the deformation γ_0 = 0.01.

$$|\eta^*| = \frac{G^*}{\omega} \quad (4)$$

$$\eta^* > \frac{F_\tau T_{min}}{\gamma_0 \omega} \quad (5)$$

where G* is complex moduli, and ω is the angular frequency.

- Inertia boundary

$$G > \frac{I F_\tau}{F_\gamma}\omega \quad (6)$$

$$F_\gamma = \frac{R_{mean}}{R_{outer} - R_{inner}} \quad (7)$$

where F_γ is conversion factor (displacement to deformation), and minimum instrument inertia I = 0.000001829 ($N{\cdot}m{\cdot}s^2$).

$$\eta^* > \frac{G^*}{\omega} = \frac{I F_\tau}{F_\gamma}\omega \quad (8)$$

Supplementary Figures and Discussion

Figure S1 shows the minimum boundaries of shear viscosity η, complex viscosity η^*, modulus G, and instrument inertia effect. The measured shear viscosity η is far above minimum boundary line, therefore it is trustworthy. While few points at the beginning of frequency sweep are below G_{min} = 0.00917 Pa (Fig. S1B), thus should be excluded, as well as the corresponding η^* points in Fig. S1A. The effect of instrument inertia in oscillatory shear rheology measurements with 3.5 wt% dispersion is also shown in Figure S1B. The dynamic modulus (G', G") in a log-log plot evolve in a manner typical for soft biomaterials at angular frequency $\omega \leq 10$ rad/s, whereas the storage modulus increases slightly with angular frequency. In the contrary, at angular frequencies $\omega > 10$ rad/s the viscous modulus and the storage modulus increase more substantially. The same applies to η^*, all η^* points below instrument inertia boundary should not be included. It would be

prudent to carefully evaluate future high frequency rheology measurements with CNC liquid-crystal dispersions for inertia.

Figure S2 shows the detail structural development of 3.5 wt% CNC with 4, 6, and 8 wt% 20 kDa PEG dispersion. Tactoid volume (V) is plotted against aspect ratio (R/r) with representative PLM microscopic images. Small volume tactoid was observed, and this effect is enhanced by a higher PEG concentration. Homogeneous tactoid was detected in 0 week dispersions with 4 and 6 wt% PEG, and cholesteric tactoid was observed after a week. While for dispersions with 8 wt% PEG, cholesteric tactoids only formed after 4 weeks. In addition, the tactoid size was smaller at high polymer concentration.

Figure S3 shows the p/2 evolution in all tactoid forming samples (pure 3.5 wt%CNC, CNC and 20 kDa PEG mixtures). p/2 is plotted against aspect ratio of the major and minor radius of tactoid. The pitch value slightly decreases with time and growth of tactoid size. It is interesting to note that even though no tactoid is observed in 0 week samples without 20 kDa PEG addition, the p/2 values measured at 1 week and 4 week are the same as the p/2 in pure CNC dispersion. We propose that the decrease of p/2 can be attributed to slight charge loss on CNC surface over time. The slight depletion attractive force induced by 20 kDa PEG can only hamper tactoid growth, not tactoid formation. Figure S4 shows PLM photos of 3.5 wt% CNC with 6/8 wt% 200 kDa PEG addition. Same as 4 wt% PEG addition, the samples with higher PEG concentrations present only nematic phase. A transient stability has been observed in the samples over time, which is in line with samples of 3.5 wt% CNC with 4 wt% 200 kDa PEG addition.

Figure S5 shows the storage modulus G' and loss modulus G" evolution of 3.5 wt% CNC with 4, 6 and 8 wt% 20 kDa and 200 kDa PEG addition. Both modulus in all mixtures increased over time, confirming that more structures are formed. The dispersions with 20 kDa PEG remained as viscoelastic liquid (G" > G') and showed higher G' and G" than pure CNC dispersions, proving that the induced depletion attractive forces enhance mechanical strength. Whereas 200 kDa PEG turned the dispersions from liquid (G" > G' in fresh samples, Fig. S5 D) into gel (G' > G" in 1-week and 4-week samples, Fig. S5 E,F) during aging.

Figure S6 shows the I(q) vs q of pure 3.5 wt% CNC (A), 3.5 wt% CNC with 4, 6 and 8 wt% 20 kDa PEG at (B); 3.5 wt% CNC with 4, 6 and 8 wt% 200 kDa PEG (C) at 0, 1 and 4 week. The difference between the time points are very difficult to tell, therefore we conducted Lorentz-corrected plot in the manuscript. Pure 4 wt% 20kDa and 4 wt% 200 kDa PEG solutions were also examined (D). The I(q) of pure polymer solutions are very low and confirms that the polymers don't form structure.

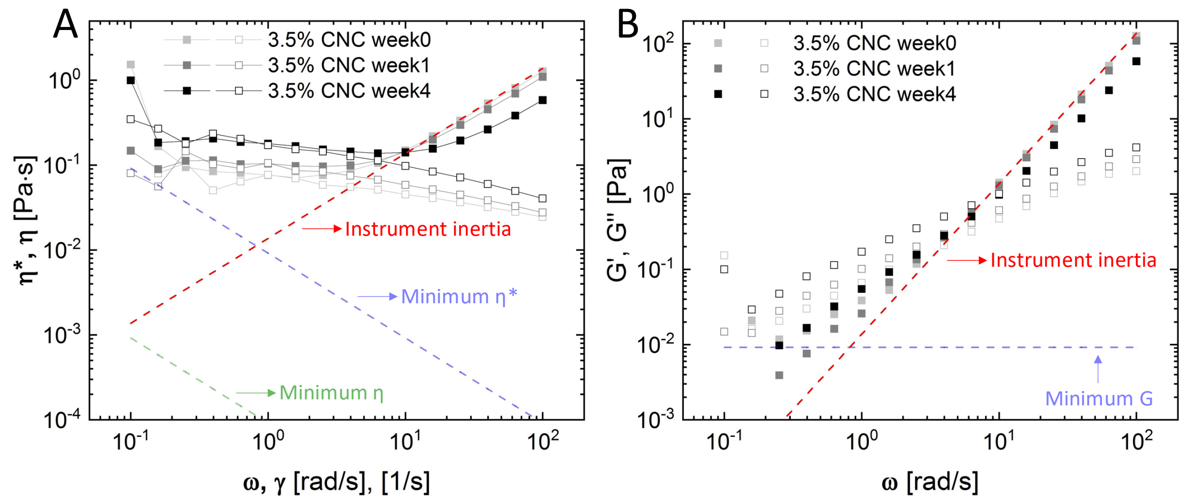

Figure S1: Minimum torque and instrument inertia boundary in pure CNC liquid-crystal dispersion measurements

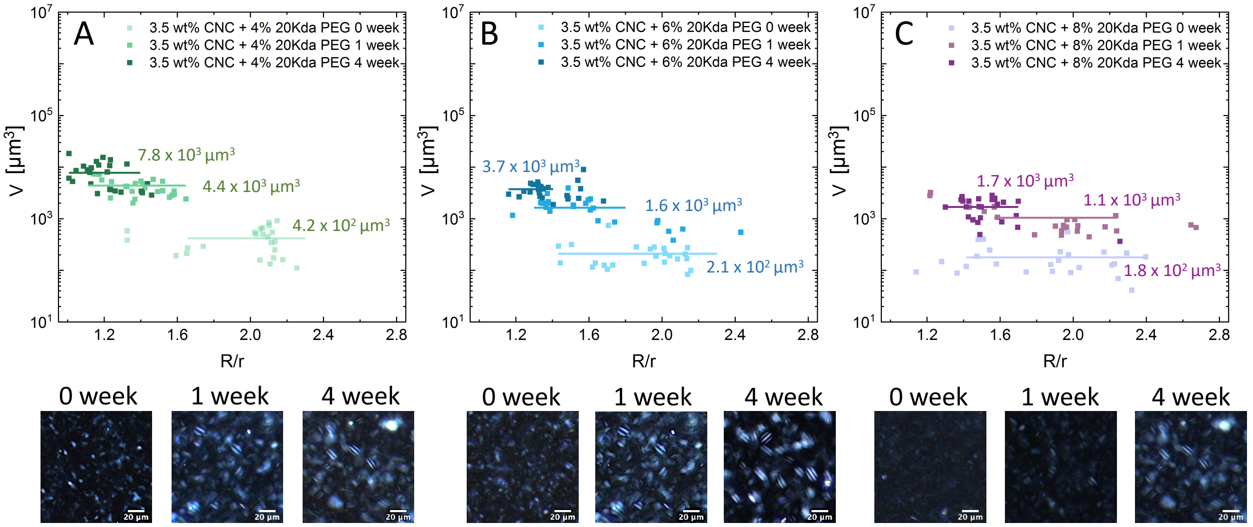

Figure S2: Development of tactoid morphology (Droplet volume vs. Aspect ratio) over time of 3.5 wt% CNC with 4 wt% 20 kDa PEG (A), 3.5 wt% CNC with 6 wt% 20 kDa PEG (B), and 3.5 wt% CNC with 8 wt% 20 kDa PEG (C). Straight lines indicate average tactoid volumes as well as span the corresponding aspect ratio ranges

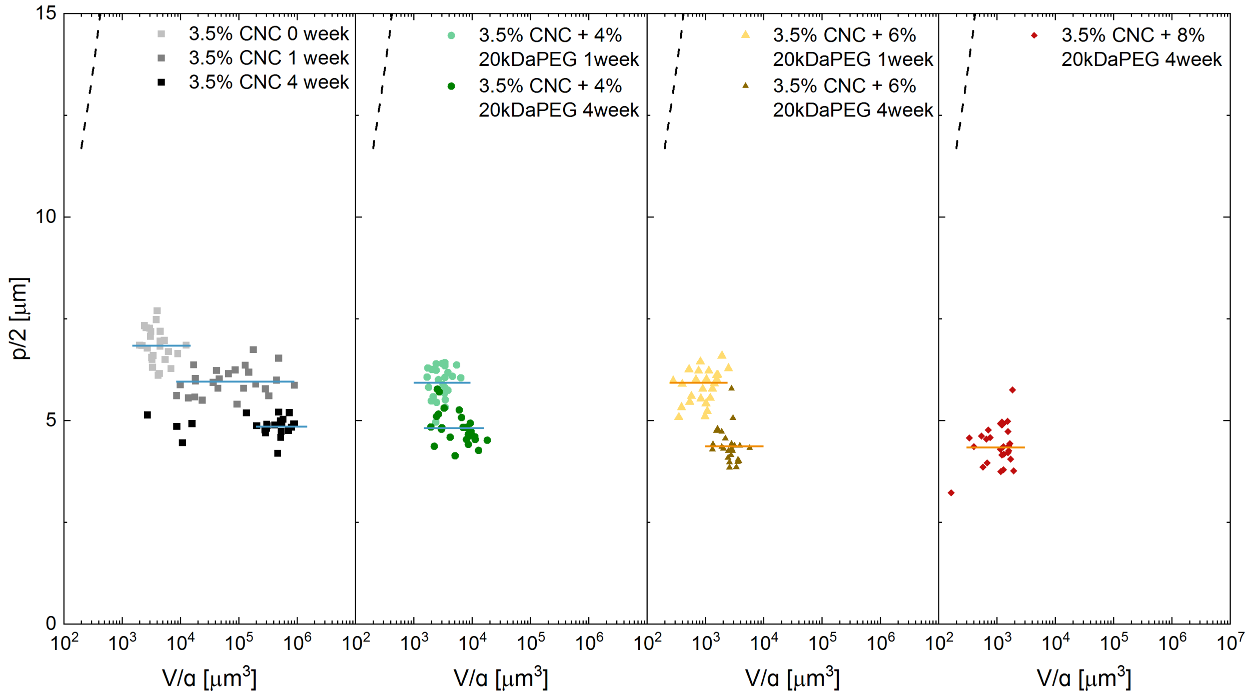

Figure S3: Pitch evolution against V/α (V denotes tactoid volume, α = R/r: aspect ratio) over time of 3.5 wt% CNC and 3.5 wt% CNC with 4, 6 and 8 wt% 20 kDa PEG

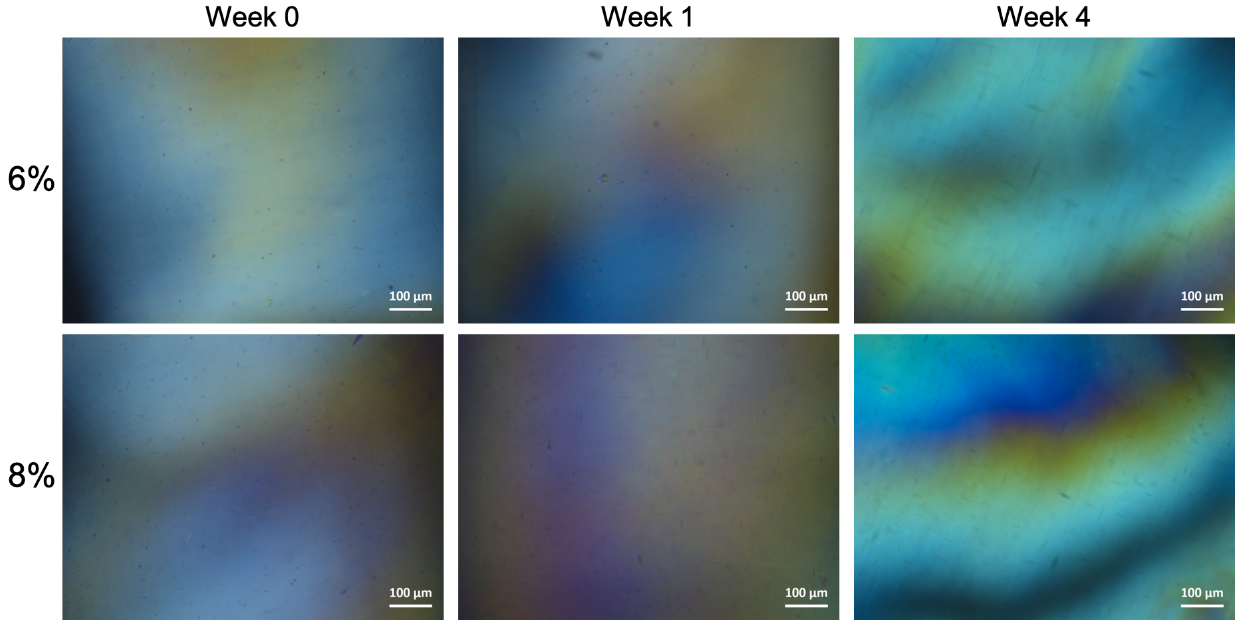

Figure S4: PLM Microscopic images of 3.5 wt% CNC with 6 and 8 wt% 200 kDa PEG

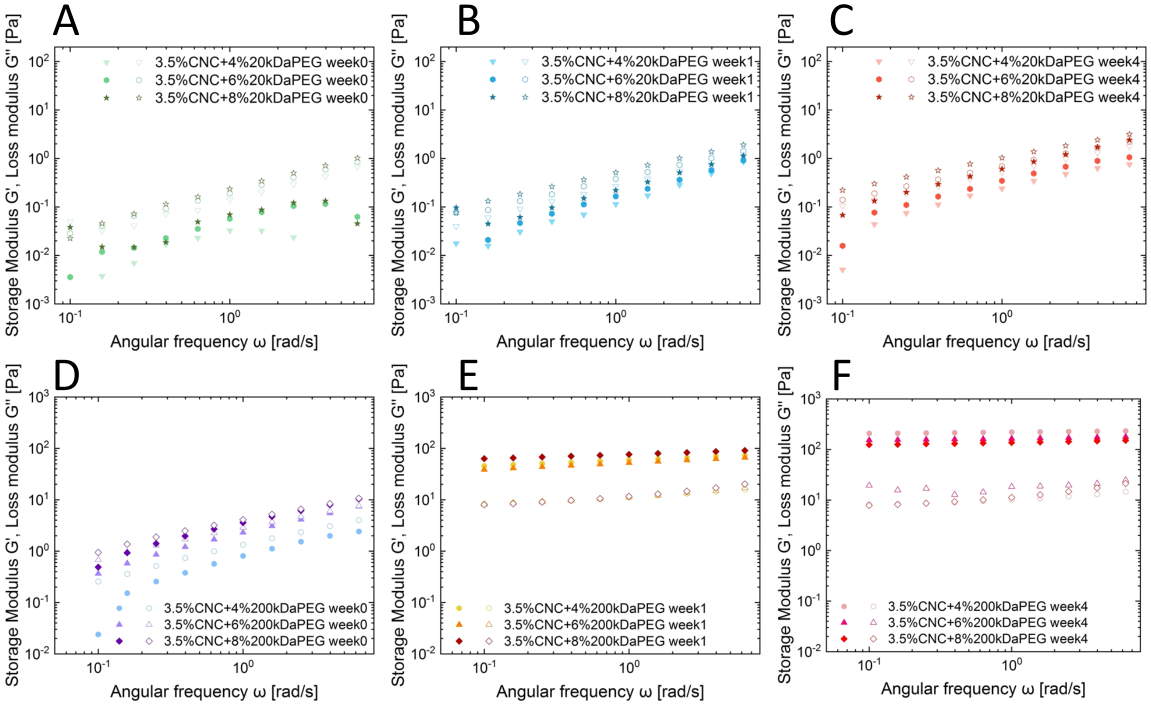

Figure S5: Evolution of storage modulus G' (solid) and loss modulus G" (empty) : 3.5 wt% CNC with 4 wt% 20 kDa PEG, 6 wt% 20 kDa PEG and 8 wt% 20 kDa PEG at 0 week (A), 1 week (B) and 4 week (C); 4 wt% 200 kDa PEG, 6 wt% 200 kDa PEG and 8 wt% 200 kDa PEG at 0 week (D), 1 week (E) and 4 week (F).

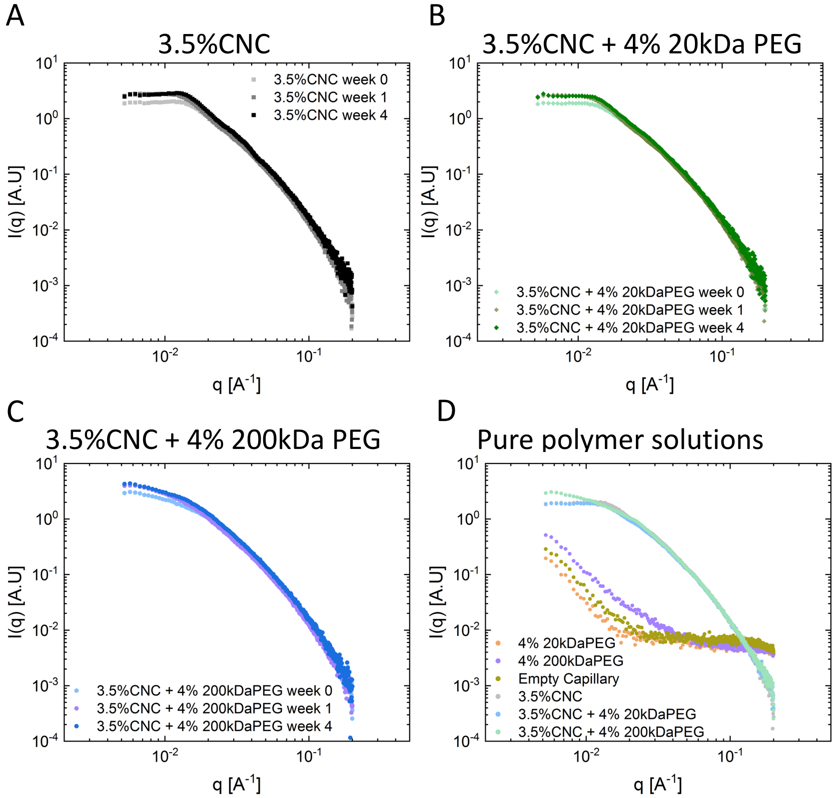

Figure S6: I(q) vs q of pure 3.5 wt% CNC (A); 3.5 wt% CNC with 4, 6 and 8 wt% 20 kDa PEG at (B); 3.5 wt% CNC with 4, 6 and 8 wt% 200 kDa PEG (C) at 0, 1 and 4 week; and pure 4 wt% 20kDa and 4 wt% 200 kDa PEG solutions, empty capillary, 3.5 wt% CNC, 3.5 wt% CNC + 4 wt% 20kDa PEG and 3.5 wt% CNC + 4 wt% 200kDa PEG at 0 week.

References

(1) Bertsch, P.; Diener, M.; Adamcik, J.; Scheuble, N.; Geue, T.; Mezzenga, R.; Fischer, P. Adsorption and interfacial layer structure of unmodified nanocrystalline cellulose at air/water interfaces. *Langmuir* 2018, *34*, 15195–15202.

(2) Mezzenga, R.; Jung, J.-M.; Adamcik, J. Effects of charge double layer and colloidal aggregation on the isotropic- nematic transition of protein fibers in water. *Langmuir* 2010, *26*, 10401–10405.

(3) Bertsch, P.; Sánchez-Ferrer, A.; Bagnani, M.; Isabettini, S.; Kohlbrecher, J.; Mezzenga, R.; Fischer, P. Ion-induced formation of nanocrystalline cellulose colloidal glasses containing nematic domains. *Langmuir* 2019, *35*, 4117–4124.

(4) Zhou, J.; Venturelli, L.; Keiser, L.; Sekatskii, S. K.; Gallaire, F.; Kasas, S.; Longo, G.; Knowles, T. P.; Ruggeri, F. S.; Dietler, G. Environmental Control of Amyloid Polymorphism by Modulation of Hydrodynamic Stress. *ACS Nano* 2020, *15*, 944–953.

(5) Usov, I.; Mezzenga, R. FiberApp: an open-source software for tracking and analyzing polymers, filaments, biomacromolecules, and fibrous objects. *Macromolecules* 2015, *48*, 1269–1280.

(6) Ewoldt, R. H.; Johnston, M. T.; Caretta, L. M. *Complex Fluids in Biological Systems*; Springer, 2015; pp 207–241.

(7) Radtke, T.; Böni, L.; Bohnacker, P.; Maggi-Beba, M.; Fischer, P.; Kriemler, S.; Benden, C.; Dressel, H. Acute effects of combined exercise and oscillatory positive expiratory pressure therapy on sputum properties and lung diffusing capacity in cystic fibrosis: a randomized, controlled, crossover trial. *BMC Pulmonary Medicine* 2018, *18*, 1–12.
